# Supplementary material for: Synchronous timing of return to breeding sites in a long-distance migratory seabird with ocean-scale variation in migration schedules
Source: Mov Ecol. 2024 Mar 22;12:22. doi: 10.1186/s40462-024-00459-9 (PMC10960466; doi:10.1186/s40462-024-00459-9)
Supplement: Supplementary file 1 — Additional file 1. Geolocation data processing, figure S1 (summary of timing data), table S1 (posterior probailities) and figure S2 (slope estimates of the duration of period). [file 40462_2024_459_MOESM1_ESM.pdf]

Supplementary material

Synchronous timing of return to breeding sites in a long-distance migratory seabird with ocean-scale variation in migration schedules

Rob SA van Bemmelen, Børge Moe, Hans Schekkerman, Sveinn Are Hansen, Katherine RS Snell, Elizabeth M Humphreys, Elina Mäntylä, Gunnar Thor Hallgrimsson, Olivier Gilg, Dorothée Ehrich, John Calladine, Sjúrður Hammer, Sarah Harris, Johannes Lang, Sölvi Rúnar Vignisson, Yann Kolbeinsson, Kimmo Nuotio, Matti Sillanpää, Benoît Sittler, Aleksandr Sokolov, Raymond H.G. Klaassen, Richard A. Phillips & Ingrid Tulp

Geolocator data processing

Sunrises and sunsets were calculated from light measurements using the function `twilightCalc` from the R-package `GeoLight` [1] at a light threshold value of 10 (arbitrary light units, BAS/Biotrack models) and 2 (lux, Migrate Technology models). As Arctic Skuas spend the non-breeding period in open habitat (at sea), light transitions at twilight were rarely shaded, and so most twilights could be assigned automatically. Remaining outliers (twilights considerably earlier or later than surrounding twilights) were identified visually by plotting date against time of sunset and sunrise and were replaced by interpolated values using a local regression (LOESS) smoother. Locations were calculated for each noon and midnight for a sequence of potential sun angles, in steps of 0.5°. By visual inspection of the resulting tracks, we selected the sun angle resulting in a good fit of locations to the shape of continent land masses and a match in latitude estimates before and after each equinox. On this basis, the most appropriate sun angles for individual tracks were from $-1°$ to $-3°$ for BAS/Biotrack loggers and $-4.5°$ to $-6.0°$ for Migrate Technology loggers. Mean error in position estimates for this method is typically ± 185 km for flying seabirds [2], but can be much larger close to the equinoxes, which coincided with autumn and to a lesser extent spring migration in the tracked skuas.

Timing of migration and breeding

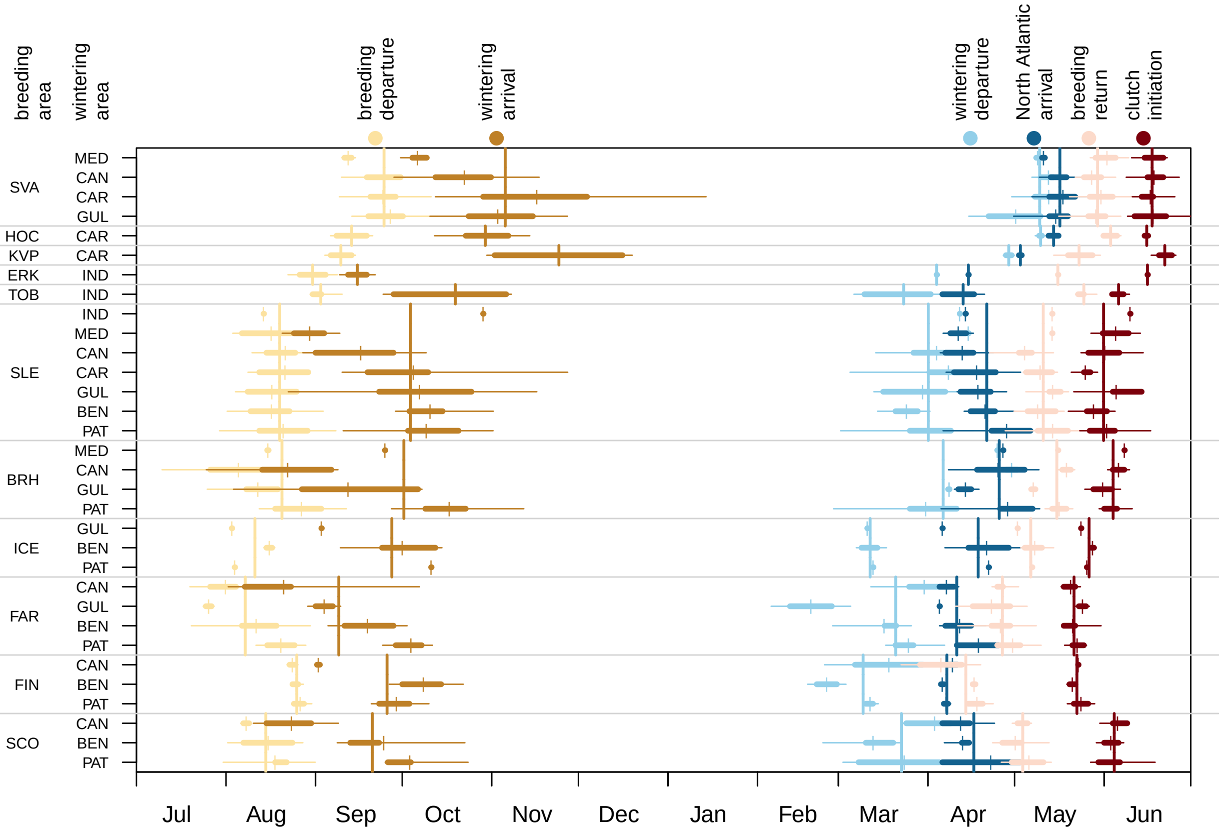

Figure S1. Summary of timing data per breeding and wintering area, not corrected for repeated tracks, of Arctic Skuas tracked from breeding areas between East Greenland and West Siberia to wintering areas in the Atlantic. Vertical lines show the mean, thick horizontal lines show the 25-75 interpercentile range and thin horizontal lines extend to the 5-95 interpercentile range. Breeding areas on the y-axis are ordered from high (SVA) to low (SCO) latitude. For birds breeding in Russia (TOB and ERK), 'arrival to the North Atlantic' refers to arrival to areas north of 35°N. For abbreviations of breeding and wintering areas, see Fig. 2.

Net **carry-over effect of wintering area**

Table S1. Posterior probability that the difference in timing or duration between two wintering areas when originating from the same breeding area is more extreme than 0 in Arctic Skuas tracked from breeding areas in the North Atlantic to wintering areas in the Atlantic. Posterior probabilities of 0.1 and lower are highlighted in red, with more intense red for lower values.

breeding area	wintering areacomparsion	breeding departure	wintering arrival	wintering departure	North Atlantic arrival	breeding return	clutch initiation	autumn migration	wintering period	spring migration	time in North Atlantic	pre-laying period	breeding period
SVA	CAR vs. GUL	0.43	0.01	0.00	0.33	0.31	0.16	0.01	0.44	0.00	0.37	0.06	0.33
	CAN vs. GUL	0.27	0.09	0.01	0.44	0.24	0.34	0.09	0.01	0.00	0.18	0.44	0.43
	CAN vs. CAR	0.31	0.00	0.42	0.41	0.10	0.30	0.00	0.00	0.20	0.24	0.03	0.42
NOR	BEN vs. PAT	0.06	0.44	0.10	0.04	0.02	0.04	0.23	0.24	0.38	0.30	0.48	0.43
	GUL vs. PAT	0.09	0.08	0.36	0.00	0.20	0.29	0.12	0.05	0.02	0.01	0.07	0.14
	CAR vs. PAT	0.36	0.34	0.03	0.02	0.08	0.00	0.38	0.08	0.00	0.06	0.34	0.31
	CAN vs. PAT	0.04	0.00	0.07	0.00	0.00	0.35	0.00	0.00	0.00	0.03	0.00	0.07
	GUL vs. BEN	0.44	0.16	0.09	0.20	0.20	0.04	0.07	0.03	0.02	0.08	0.13	0.23
	CAR vs. BEN	0.21	0.40	0.01	0.31	0.42	0.19	0.22	0.04	0.00	0.19	0.36	0.29
	CAN vs. BEN	0.50	0.00	0.01	0.04	0.14	0.10	0.00	0.00	0.00	0.15	0.01	0.09
	CAR vs. GUL	0.25	0.27	0.09	0.40	0.29	0.01	0.30	0.50	0.12	0.34	0.08	0.11
	CAN vs. GUL	0.44	0.00	0.20	0.18	0.03	0.23	0.01	0.02	0.07	0.23	0.14	0.02
	CAN vs. CAR	0.19	0.00	0.25	0.17	0.11	0.01	0.01	0.04	0.46	0.45	0.01	0.26

Table S1. Posterior probability that the difference in timing or duration between two wintering areas when originating from the same breeding area is more extreme than 0 in Arctic Skuas tracked from breeding areas in the North Atlantic to wintering areas in the Atlantic. Posterior probabilities of 0.1 and lower are highlighted in red, with more intense red for lower values.

breeding area	wintering areacomparsion	breeding departure	wintering arrival	wintering departure	North Atlantic arrival	breeding return	clutch initiation	autumn migration	wintering period	spring migration	time in North Atlantic	pre-laying period	breeding period
FAR	BEN vs. PAT	0.06	0.10	0.11	0.08			0.29	0.39		0.23		
	CAN vs. PAT	0.00	0.00	0.32	0.01			0.01	0.00		0.03		
	CAN vs. BEN	0.05	0.00	0.02	0.19	0.36	0.40	0.01	0.00	0.05	0.10	0.34	0.37
SCO	BEN vs. PAT	0.28	0.24	0.08	0.04	0.16	0.38	0.34	0.44	0.14	0.00	0.13	0.44
	CAN vs. PAT	0.08	0.00	0.20	0.04	0.31	0.43	0.01	0.00	0.06	0.00	0.37	0.14
	CAN vs. BEN	0.19	0.01	0.01	0.46	0.32	0.32	0.02	0.00	0.00	0.17	0.22	0.16

***Strength* of the carry-over effect: slope estimates**

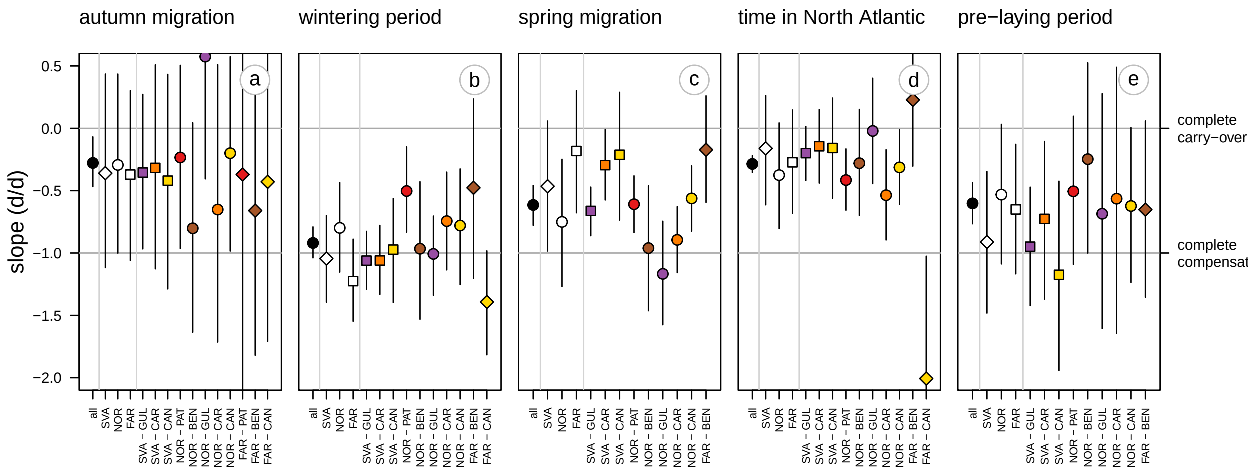

Figure S2. Slope estimates of the duration of periods as a function of the timing of their start in Arctic Skuas tracked from breeding areas in the North Atlantic to wintering areas in the Atlantic (Fig. 5). Steepness of the slopes indicates the strength of carry-over effects: they show to what extent advances or delays at the start translate to the timing of the end of each period. For example, a slope of 0 indicates that every day delay at the start of a period translates in a day delay at the end of the period, and a slope of -1 indicates that every day delay is compensated so that the duration of the period gets one day shorter. Estimates are given at three levels, which are separated by grey vertical lines: 1. across all breeding and wintering areas, 2. for each of the three breeding areas and 3. for each combination of breeding and wintering area. Error bars are 95% HDIs.

Literature

1. Lisovski S, Hahn S. 2012 GeoLight - processing and analysing light-based geolocator data in R. *Methods in Ecology and Evolution* **3**, 1055–1059. (doi:10.1111/j.2041-210X.2012.00248.x)

2. Phillips RA, Silk JRD, Croxall JP, Afanasyev V, Briggs DR. 2004 Accuracy of geolocation estimates for flying seabirds. *Marine Ecology Progress Series* **266**, 265–272. (doi:10.3354/meps266265)
